# Parent-Researcher Perspectives on Role Intersectionality Related to Autism Research

**DOI:** 10.3389/fresc.2022.718398

**Published:** 2022-03-14

**Authors:** Laura J. Hall, Natalie A. Hoxie, Gretchen S. Grundon, Yulian N. Cordero

**Affiliations:** Department of Special Education, San Diego State University, San Diego, CA, United States

**Keywords:** parent-researcher, autism, co-researcher, autoethnography, parent-professional, parent participation

## Abstract

Although parents of children with autism who are also experts in a related profession have been instrumental in shaping current practices, there is little known about parent-researchers and the benefits and obstacles to including individuals with these intersectional identities on autism focused research teams. The following study used collaborative autoethnographies from three parent-researchers hired for a large scale, federally funded project. The parent-researchers, and co-authors, collaborated on all phases of the reported study. Common themes generated from the shared perspectives included: prioritizing children and professional sacrifices; professional training as an asset for parents; potential bias toward parents in professional contexts; assets as parent-professionals; and obstacles for maintaining intersectional roles of parent-professionals. Recommendations for autism researchers and considerations for employing and supporting parent-researchers are discussed.

## Introduction

Following interviews with international disability rights leaders, James Charlton (1) published the book, *Nothing About Us Without Us: Disability Oppression and Empowerment*, where he advocates for elevating the perspectives and voices of individuals with the lived experience of disability. He describes the disability rights movement that began in the 1960's and 1970's that has the aim of changing systems to incorporate people with disabilities into the decision-making process because it is recognized that “the experiential knowledge of these people is pivotal in making decisions that affect their lives” (p. 17). Historically, individuals with such lived experiences have been leaders who have contributed to our knowledge and understanding of how the field can best support and enhance the quality of life for individuals and families (2). The following article focuses on the perspectives of three parent-researchers hired for a federally funded project focused on high school^1^ students with autism.

There are numerous expert professionals who are parents of children with autism that have made major contributions to the field and to the quality of life for many individuals and families. For example, Bernard Rimland (3) a parent, advocate, and research psychologist wrote the book, *Infantile Autism*, which shifted the focus of our understanding about the cause of autism from poor parenting to neurobiology; Catherine Maurice (4) a journalist and parent who chronicled her experience obtaining a diagnosis and effective science-based interventions for her children with autism in *Let Me Hear Your Voice*; Roy Richard Grinker (5) a parent, anthropologist, and author of *Unstrange Minds: Remapping the World of Autism*, who focuses his work on how culture and context influences our definitions and understanding of autism; and Lori Unumb, parent, lawyer, and autism advocate whose efforts have resulted in legislation in support of the provision of services by insurance companies. Parents and grandparents also have been instrumental in providing the fiscal and other resources for the development of currently renowned research and educational organizations such as the UC Davis MIND institute, the Princeton Child Development Institute, Autism Speaks, and the Organization for Autism Research to name a few. Even with these well-known examples of the impact of parent-professionals there is little known about the experiences of parent-professionals who are part of autism-focused research teams. In addition, the contribution that can be made to autism research by hiring individuals with the intersectional perspectives of parent and professional is not well-known, and individuals who hold these perspectives are not necessarily sought out to fill positions on research teams.

Morris et al. (6) argue that it is crucial to involve families in all phases of disability research because they are uniquely positioned to consider which areas of research inquiry are most relevant and potentially impactful on their lives or the health and wellbeing of children with disabilities. These authors also argue that family involvement may shift the power over the research agenda and in the research process (6). This shift in power not only benefits the family members involved in the research, it can simultaneously provide the researchers the opportunity to experience the ways a strong community partnership can strengthen research efforts (7). Conducting research collaboratively with parents modifies the hierarchy between researcher and participant and can add depth and texture to our understanding of the research process and research outcomes (8). Hackett (8) who has conducted co-research with parents in northern England commented that, “Few studies have positioned parents as co-researchers” (p. 483).

In their 2016 scoping review of the literature, Shen et al. identify the benefits and challenges of engaging parents as co-researchers in health research. They identified ten studies and concluded that involvement of parents as co-researchers resulted in the development of more appropriate and sustained intervention, facilitated research participation, and increased the sense of empowerment by parent researchers. In only half of the reviewed studies, parents acted as the facilitators of the intervention or were involved in dissemination of findings, and even fewer studies had parents participate in the development of research questions (9). There were two studies in this review focused on disability (10) and one study included parents of individuals with intellectual disability as co-researchers (11). In this study parents were recruited and trained as co-facilitators of focus groups held throughout Ireland. Co-facilitators were interviewed about the impact of their co-researching. They reported that they served as role models for parents, that their involvement encouraged frank discussion in the focus groups; that they were instrumental to informing the research team about the importance of selecting locations and meeting times that were convenient for parents; and their involvement had the benefit of empowering the parent-researchers. Parents involved in the studies reviewed by Shen et al. (9) were not necessarily professionals in the health services field and some parents experienced frustration, disappointment, and powerlessness as a result of their lack of understanding of research logistics.

This article aims to provide a better understanding of the benefits for, and obstacles to, stakeholder involvement in autism research. The data source used is the parent-researcher's (who are co-authors) shared experiences gathered through the method of collaborative autoethnography. Autoethnography is a form of self-narrative derived from the field of anthropology that is used for cultural analysis and interpretation (12). Autoethnography is increasingly used by educational researchers as a means of addressing social problems and has been disseminated for publication in first-tier, peer-reviewed journals (13). Autoethnographies can serve as a vehicle for empowering marginalized voices using a method that includes opportunity for participants to reflect upon and consider how their perspectives are represented (13, 14).

Collaborative autoethnography enables researchers to benefit from simultaneous self and collective analysis resulting in collaborative exploration, power-sharing among co-authors, deeper learning about self and others, and community building (15). Autoethnography can be used to explore the interplay of social categorizations that form an individual's identity such as race, gender, family position, religious affiliation, and profession. For example, Hannon (16) used autoethnography to describe the intersectionality of a black school counselor and father of a student with autism. In their collaborative autoethnography three women of color describe the intersectionality of race, gender and role as a university graduate student (14). The purpose of this article is to describe and analyze the intersectionality of a parent of a child with autism and the role of professional and researcher conducting autism research. Collective autoethnography was the method selected for this study because it provides each parent-professional the opportunity to share their voice about the intersectionality of these roles from their perspectives and empowers the parent-researchers as equal co-authors.

## Method

### Co-authors

Each of the participating co-authors was hired to work on a research team at one of the three participating Center on Secondary Education for Students with Autism spectrum disorder (CSESA) sites conducting a clustered randomized controlled trial (CRCT) of a multi-component intervention for high school youth with autism and a post-graduation follow-up study. CSESA was funded by two grants from the US Department of Education Institute of Education Sciences to (a) coach school personnel as they implement project developed curriculum that address four key areas of support for youth with autism (social competence, reading comprehension, transition and independence and behavior) (17) and (b) evaluate post high school outcomes for study participants. The first author is a White, female, university Professor who was one of the CSESA site Co-PIs with a history of conducting research focused on autism. During an advisory meeting held prior to the start of the CRCT study, a researcher who had completed a similar multisite project stated that one of the challenges in that project was effective communication with families and creating the supports needed to ensure assessment measures were collected. In response, Natalie Hoxie, a woman of Asian and European descent was hired. The decision to hire this parent-researcher was without regret as she was able to share comments at recruitment sessions, such as “I wish there had been a project like this in place when my child was in high school.” and “You can trust these people,” that resonated with families.

One of the project components involved a program of information and support sessions for parents entitled, Transitioning Together (18), that, at this site, was led by a Marriage and Family Therapist and a representative from the high school staff. The MFT requested that Yulian Cordero, who is bilingual and Mexican American, also work with the parent groups in order to complete her supervised hours for her MFT license. Her talents quickly became apparent and Yulian was asked to work on the research in another capacity that involved conducting pilot interviews with parents.

When the CSESA project was awarded the funds for the 2-year follow-up study the cross-site team discussed that they valued the contribution made by parents and that each site would aim to hire parent-researchers for the teams. Gretchen Grundon, a White woman, made the decision to leave employment with an agency so she could devote more time to her daughter with autism and contacted the first author to arrange for part-time employment. She and the other two parent-researchers in this study were hired for the follow-up study at one site and conducted the vast majority of the parent interviews, a main source of data for the CSESA follow up study.

### Procedures

Guidelines for use of collaborative autoethnography as a tool for researchers developed by Chang et al. (12, 15) were followed for this article. The following steps for this qualitative research method were followed: (1) Identify a research focus and what to study; (2) Collect data in the form of personal memory data or autoethnography writing; (3) Conduct data analysis by reviewing and coding the data into topical groupings or categories; (4) Identify themes and reconnect with support from data and (5) Interpret the data by describing the meaning of the data, how the information advances understanding of the topic and connecting findings to the existing literature. Chang et al. (15) add that the iterative and dynamic dialog that occurs throughout the collaborative autoethnography research process means that the researchers are continually engaged in collective meaning-making.

Following approval by the university Institutional Review Board, the co-authors met to collaboratively design the study and identified that the focus would be on the intersectionality of a parent of a child with autism and researcher. The research method of autoethnography was selected. Relevant articles for the literature review were placed in a google drive for comment by all authors and the introduction was collaboratively written. The co-authors discussed the current literature and collaboratively developed the probe questions or topic areas to be used as a guide for the autoethnographies so that there would be consistent content across the three autoethnographies and these included: Professional experience (e.g., choice of profession; were there obstacles, biases or situation where your role as mother influenced your decisions or actions?); Parent of a child with autism (e.g., type of support; obstacles, biases, or situations where your role as a professional influenced decisions); Education (e.g., background; experiences) and Research environment (e.g., added value of both roles; challenges and benefits, experiences with the Center on Secondary Education for Students with Autism project). Each of the second through fourth co-authors wrote their autoethnography independently and sent it to the first author who is not a parent of a child with autism. The procedures used to conduct a thematic analysis were guided by the recommendations of Nowell et al. (19) to establish trustworthiness that incorporate the six recommended steps of thematic analysis taken from Braun and Clarks (20) to (1) familiarize yourself with the data, (2) generate initial codes, (3) search for themes, (4) review themes, (5) define and name themes and (6) produce the report. The first author edited the submissions for clarity and flow and returned them to each author to review and reconfirm consent to share the content. Then the first author (a) became familiar with the content, (b) generated initial codes and marked the comments into color coded content for identified codes, and (c) searched for themes. All three coded autoethnographies were (d) sent to, and vetted by, the co-authors to read and further define the themes. This form of member checking to establish the fit between the respondents' views and presentation of the data establishes credibility of the findings (19). All authors (e) then met through Zoom to discuss the impact of the stories, compare themes, identify sub themes from the autoethnographies and confirmed the themes by each of the co-authors for accuracy (19). Any disagreements in the interpretation of the content or generation of themes were discussed with all respondents and co-authors until consensus was reached. Following another review of the themes by each of the three parent-researchers, the four co-authors met to organize the discussion to describe common themes, implications of the shared experiences, and recommendations for autism researchers. The meetings with all co-authors were recorded and reviewed to confirm the final themes.

## Natalie

### Personal and Educational History

I did not get married until my mid 30's and had my first of three children at 35. When my son was 18 months, I noticed a profound change in his ability to communicate both verbally and non-verbally. He was assessed by a developmental psychologist and speech therapist and diagnosed with severe autism and developmental and communicative delay. At ten he developed refractory epilepsy and began having tonic and clonic seizures. I had already had a career in healthcare for 15 years. I had always planned on quitting my job to take care of my children, at least until my children were school age. I am grateful that this was our plan because I could not have returned to work and provided my son with the intensive early intervention services he needed. I would have had difficulty securing childcare for a child with a severe developmental disability. I know of many women who were forced to leave a career that they absolutely loved to help their child with a disability. They simply did not see an alternative and this is quite sad.

Staying home was the only way my husband could work full time and my son could attend his healthcare appointments and be available for intensive, in-home, early intervention. Managing the health/social service/educational needs of a child with a severe disability is akin to running a small business, for which you are not paid and are responsible for keeping all parties accountable. From as early as 18 months I had to take legal action to secure behavioral services and I learned very quickly that I needed to make it my “profession” to understand everything about the health/social services/ educational services my son would be interfacing with. I needed to know what services were available, what he needed, what he was legally entitled to, and the laws and policies that governed these systems.

If I wanted to return to work, my husband would have helped me find a way but we both knew I needed to make sure our son had access to what he needed, and I was the right person for the job. I have been told that I can be quite persistent and creative. I have always believed that I should never accept, “no” from someone who lacks the authority to give me a “yes.”

In 2009, with three children under ten years of age, I needed to do something like go back to work or school just to focus on my own health and intellectual growth so I went back to school for a master's degree in special education so that I could better meet the needs of my child. I was able to meet this objective, and, in that process, I stumbled into opportunities that I had not previously considered, such as becoming a Board Certified Behavior Analyst (BCBA), lecturer, and researcher. I really enjoy the university setting where I am currently a lecturer. I enjoy being around young, energetic, optimistic young educators. Based on their evaluations of me, they seem to appreciate what I bring to the table. I have recently started a doctorate program in education leadership and am focused on improving special education practice and increasing inclusive opportunities for all students.

### The Intersectionality of Parent and Professional

Prior to having children, I worked in the healthcare field as a practitioner and an administrator. I took care of patients in all stages of life and then recruitment, staffing, marketing, and contracting with health care agencies. I also was involved in contracting for and placing speech and occupational therapists in public schools.

I never intended to pursue a career in special education or autism. You could say that I did not go looking for autism, it came looking for me. I started off my master's to understand special education from the inside out, so I could learn how to fight for my child. I needed to better understand teaching methods, programs, the language and culture of special education, the power structure in my school district, and the laws governing special education. Initially, this was a mission to help my son.

Once I took some classes, I quickly became interested in behavior analysis. My own experience as a healthcare provider, along with being a parent who saw the benefits to my own child, allowed me to collaborate with parents in a meaningful way. I was later offered a co-teaching position in special education at the university level and, although I had no teaching experience, I was paired with a very welcoming co-teacher, have a very supportive mentor, and have enjoyed success as a part-time lecturer and coordinator of a practicum for students pursuing certification in behavior analysis. What started out as solely an endeavor to help my child ended up opening doors of opportunity that I had not previously considered. These opportunities enabled me to grow professionally without interfering with meeting my son's needs or disrupting my family life.

Although I am an exceptional advocate, and could probably make a good living doing advocacy, as a mother, I know doing this full time would take its toll on me emotionally. Instead, I prefer to coach parents to advocate for their own children as advocating for your own child can be very rewarding and educational. I also prefer to do what I can to influence young teachers and encourage them to welcome parent involvement and see it as a positive. I also like to help young teachers see the “long game” when selecting goals for their students and encourage them to write goals with social value that can last a lifetime.

During graduate school, and even in my current research, I often do not mention that I am a parent of a child with a disability. I believe that there is this perception that parents cannot be objective about their own child, have little to contribute, and don't know as much as academics who engage in educational research. While the program director welcomed me to the autism program, the department chair at the time met with me and said, “If you are not a teacher, why are you here?” I replied, “I suppose it is because I know that being a teacher is just one of many ways to help a child with a disability and to really help a child with a disability it is a good idea to understand the programs they participate in every day to the best of your ability.” I can recall that in some of my classes, I did not mention that I was a parent for weeks and was able to hear the true attitudes of young teachers and even professors as they bemoaned the “high profile” parent who was currently making their life miserable. I was not surprised. When I did finally disclose that I was a parent, I tried to introduce a new perspective to the conversation, and I like to think I helped some of these young teachers see parents in a different and more positive light.

### Parent-Professional as Co-researcher

My professional and research choices reflect my interests and the issues I care about. Having a child with a severe disability has taught me more than I ever thought I would ever know about social services, special education, and healthcare bureaucracy and advocacy. The choices of projects I take on also are dictated by my availability and the demands of raising a child with a severe developmental disability.

#### Assets and Challenges

Having an advanced degree in education, along with being a parent, adds to my assets as a researcher because higher education, among other things, teaches you how to engage in critical thinking and conduct research. By being as current as I can with evidence-based research, I know what can threaten the validity of research outcomes and know what to avoid. I also believe that thinking about social value whenever you conduct research is essential to answering questions that are useful to the population you are trying to help. I often read a lot of articles in journals and say to myself “interesting, but so what?” which is my way of saying that a study can have significant results but no social value.

When I worked in healthcare, there were very strict codes of conduct to protect private information and maintain professional, ethical practice. The healthcare field is also focused on procedure so I, and the other parent researchers I work with, developed systems to ensure that all of our responsibilities and interviews followed a procedure.

I think being a parent helps but being a parent with a child with disability forces you to interface with professionals and agencies that parents of typical children never encounter. Understanding the language and the policies and procedures of engaging with these entities prevents the need for a parent to explain what they mean to me as a researcher. Although most of the parents I interviewed did not know I had a child with a disability, there was a palpable sense that we knew what they meant when they explained a situation they were going through with a certain entity and that made them feel comfortable about being candid with their responses. It allowed them to go into greater detail once they knew that I understood what they were talking about.

#### Benefits of Parent-Professional Participation

There are many benefits to participating as a researcher. It was not until I was invited by my employer to do so that I even considered engaging in research. I was very moved by her confidence in me and her belief that I could contribute in a meaningful way. This helped me see myself as a researcher and added the experience to my resume. It also has helped me as I write my own dissertation. Secondarily, I have enjoyed working with other researcher-parents and have great respect for them because I know how important it is to them to not only do the work but to do it well. They are inspiring and push me to do better. I have also benefited from the parents I have interviewed and have learned from them and their endeavors to carve out a better life for their children.

Most parents I know value research and have been reading studies since their child's diagnosis at an early age. Parents do not always feel that research relates to them because autism is so diverse in its impact on individuals. A lot of research tends to be focused on, I hate this term, the “higher functioning” and a lot of parents of children who have been impacted to a greater degree by autism do not see themselves in a lot of the research. Most parents would agree that research can help you access the services your child needs. For example, the research conducted on effective interventions for children with autism by O. Ivar Lovaas at the University of California, Los Angeles formed the basis of parent special education complaints and required school districts to develop autism programs that were based on research and conferred meaningful academic benefit. These cases involving the intersection of research and parent involvement are often referred to as the Lovaas Cases.

### Barriers to Participation as a Parent-Researcher

Sometimes studies do not build in seeking out parents with higher degrees for participation in research and it is unclear to me as to why. I think there is a presumption that parents in general are not objective about their children, and that professional researchers know what research is useful to the field, and parents are just not seen as an asset. Some of the articles suggest that getting parents to participate is largely a symbolic gesture. The only reason I could guess as to why parents like us are not sought out is that there is some bias, and perhaps the belief, that a non-parent would be better at gathering information. Perhaps this question should be posed to researchers. It would, I believe, be of great benefit to researchers to ask themselves if they believe an educated parent researcher would help or harm their study and, if they believe it would be harmful, self-reflect and ask themselves if they harbor implicit biases. If researchers value what parent researchers bring to the table they will find a way to compensate them for their expertise and create a professional environment to make it happen. The barriers are pretty minimal.

### Researcher With CSESA

I never considered participating in research until the opportunity for CSESA arose. I chose to be involved after learning more about what my responsibilities would entail and the time commitment. After being offered the opportunity and gaining a better understanding of what my role would be, I enjoyed the opportunity to be involved in research at this level. I made the decision to participate once I knew that scheduling was flexible and the job could work around my schedule and the needs of my family, especially my child with special needs. I chose to be involved mostly because it was something I had never done before and I welcomed the opportunity.

CSESA allowed me to schedule when my parent and young adult meetings would be, which, as it turns out, tended to be better for the parents I was interviewing. For example, by catching them on the weekend, we were able to meet with them when they were rested and not after a long day of school or work. This flexibility, mileage reimbursement, and payment for participation in the research helped make the experience worthwhile.

CSESA allowed us to partner up with another parent researcher so that we could visit families together. This increased our comfort and safety in going into peoples' homes and allowed us to carpool. It also allowed us to interview the young adult and parent separately, which was not only more efficient timewise, but helped us to see that parental assumptions and what the young adults shared were often different. I valued this opportunity to work with two other parent researchers who were very supportive and were willing to jump in and cover if I was unable to make an appointment and vice-versa. We supported each other because we understood that, at any moment, something could happen that required our immediate attention and it was nice to have that safety net. I would like to have been more involved with selection of assessments, designing the study, and writing up the questionnaire.

Like the parents we interviewed, all of us were interfacing with the same social service agencies, healthcare systems, and had a good understanding of public education so when the parents discussed programs that they found particularly helpful or not helpful we understood what their frustrations were and could ask questions on a level that conveyed that level of understanding. This allowed them to provide more detailed information.

As a parent, it made me so proud to be in the company of so many devoted parents. It allowed me to learn about some of the grassroots organizations that parents have initiated to meet the needs of their young adults. As an educator, it affirmed my belief that there is a need to share with young teachers that healthy partnerships with parents can contribute to good student outcomes and failure to do so is a missed opportunity. As a professional, it added to the breadth of my experience and training.

## Gretchen

### Personal and Educational History

I graduated college with a psychology degree but decided that I was no longer interested in pursuing my first goal of becoming a school psychologist. The summer after I graduated, I fell into a job as a special education aide and that was it-from then on, I worked in special education. I fell in love with it and knew that it was the field for me. While working as an aide, I applied for graduate school. About 2 years later, though I had never pictured myself as a teacher, I held in my hand a special education teaching credential. In the field I have worked as a teacher, ABA supervisor and program manager for both home and school programs, parent trainer, early intervention service provider, university faculty member and I manage a small social media account and website for families who have children with special needs.

I have always loved children and always saw myself being a parent, but I never had a specific timeline in mind after I got married. I had already been in the field for many years when my daughter was diagnosed as at-risk for autism. Early on, the choice for me to become a parent was parallel to going back to school. I remember saying to myself, “I'll try for a year to have a family and if it doesn't happen, I'll go back to school to get my PhD.” Two years after I finished my master's degree, the family came, and the PhD did not. My older daughter is typically developing, and my younger daughter has a genetic condition called Xia Gibbs Syndrome. It is an extremely rare disorder that affects an individual's cognitive, physical, and behavioral characteristics. Along with Xia Gibbs, she is diagnosed with autism, ADHD, anxiety, intellectual disability, and displays a complex health and behavior profile.

The experience I had under my belt by the time I had my girls helped in getting my younger daughter identified and into services. From the day she was born, I knew that she was going to need extra support. She had a difficult entry into the world and my knowledge of early childhood special education helped me understand what red flags to watch for and how to determine when to ask for help. I was thankful for the support and connections I had in our community because of the years I had spent in the field prior to becoming a mother.

As a mother of an infant with special needs, as well as a one-year-old, I knew that working full time as a teacher was going to be too much for me. As a young married couple, we had always wanted a family, but me staying home full time with kids was never a part of our original plan. Financially it just wasn't something we thought we could make happen. But I cut back to half time as soon as my second daughter was born and, since then, every job change I have made has been due to the needs of my family.

My husband is a very hands-on father. He owns his own business so he has more flexibility in his schedule than many of the fathers I know. We also decided early on that if I was going to work only part time, I would be primarily responsible for the children, so that he could focus on his work and I would ask for support when I needed it. This model typically works well for us, but when I was in a “part time job,” actually working full time, the dynamic was often rough on both of us. We live a life of “dividing and conquering,” and in my experience, many parents often experience feelings of inequality when it comes to the balance of work, running a household and raising children, as well as also caring for aging family members. Our family is no different. Parenting both a child with special needs and a typically developing child can be very stressful at times but we continue to modify our routine to try to make the best of it. My husband also tries to stay actively involved with our daughter's programming, meets with service providers, and will follow my lead to implement strategies in the home. We strive to find a balance between attention given to our daughter with special needs and our typically developing daughter. The pandemic has put even more strain on this as our support systems have shifted dramatically in the past year. We are making it work though, albeit with a few more gray hairs and some dark circles under our eyes.

### Intersectionality of Parent and Professional

I think my role as a professional working in the field of special education completely changed when I became a mother. And then, when I became a mother of a child with special needs, it changed even more. It is hard to know what it is like to be a parent of a child with special needs until you become one and it is hard to explain what I mean by that. It's just a feeling you get when you can say to another parent, “I know what you are going through,” and actually KNOW what they are going through. For me, I think the part of my role as an educator that changed the most is the way I approach parent training and the decisions I have made about selecting and designing plans for parents to follow in their homes. I have a more realistic outlook on what we can and should expect parents to do and my skills in working with families have improved immensely over time. I used to not understand why some parents would do the things they do and would get frustrated sometimes when they would not implement something I had suggested. Now I look back on some of those things and laugh at the difficulty of interventions I had recommended to families. When I do recommend things that are hard because I know it is the family's best option, as a mother, I can say “I know this is going to be really hard. It was hard for me when I did it with my daughter, but it worked, and I know you can do it. Are you willing to try?”

After I had kids, every professional decision I made was with them in mind. I think once you become a parent, this is inevitable. You aren't just choosing for yourself anymore; you are making choices that will impact your family. And, when your child has special needs, this is even more apparent. Will this job allow me to get my child to all of her appointments and therapies? Will I have flexibility during the day to be on hold with an insurance company for 2 h if I need to be? Will I have the energy to implement a behavior plan for decreasing food selectively at dinner if I have been working all day? Will I be able to homeschool my child during a worldwide pandemic and still have enough time to get my work done without losing my mind? When your child has special needs, no other role in your life is more important.

Regarding professional decisions, I think it became more necessary for me to speak up for what I needed in my career as my daughter's needs intensified. Prior to having kids, I was your run-of-the-mill workaholic. You know the type-first one to arrive, last one to leave. I was tired all the time, but I was not good at saying, “no,” or with being okay with a quality of work that wasn't exemplary. In some ways I began to set better boundaries professionally when I became a parent of a child with special needs, but in other ways, this became more challenging as a new layer of guilt began to arise. My work families needed me, the same way our family needed the support of our therapists and providers. I was having to choose between being home with my own child who needed my help and staying at work longer to help a family who was also in crisis. This is part of the reason I had to step away from direct services positions and working intensely with families. I struggled too much to find the perfect balance.

When I was a teacher, it was extremely difficult to balance childcare, doctor appointments, and sleepless nights up with sick babies. As a young mother no one really put me down for calling out, but I felt guilty when I did. When I worked part time for a private ABA company, there was more flexibility than in teaching, but I still experienced guilt when I needed to call out to attend to my family. As the years went on, I felt like experience wise I was ready for more professional challenges, but I knew that because of my demands as a mother, I couldn't take on more. It was very hard for me when I started getting passed up by others who were willing or able to put in more time than I could. I had to learn how to always put my family first, even if it meant saying no to, or not being offered, professional promotions, or watching people younger than me, that I had trained, surpass me professionally in the workplace.

Before I had my children, I had a strong desire to pursue a Ph.D., but so far, the timing hasn't been right. It is still something I think about though. I have always worked in some capacity since having my children, aside from my initial maternity leaves, but I do not think I will work full time again while they are young, or maybe ever. I feel thankful to have the flexibility to still work a bit while being able to support my children.

### Parent-Professional as Co-researcher

Education in a science-based field has allowed me to learn about the research process. Through college and graduate school, I experienced all the roles of being involved with research, from being a subject, to being an assistant to the process, to being a more active participant in designing and conducting research studies. As a BCBA I can understand the value of research and what it contributes to the field as well.

#### Assets

Although I think that everyone brings strengths to the table, there is something to be said about parents connecting with other parents. I like to believe that parents feel comfortable with me and know that they can be themselves when I am with them. Over the years I have worked with many families, and thus gained a wide breadth of experience of how different families operate, culturally, economically, and socially. Additionally, because I've worked with different types of families, I've learned a great deal about services available in our community, as well as in surrounding communities. I typically feel immediately comfortable when connecting with new families, regardless of the situation because I am used to the process of working with families on a professional level and have worked with families in a variety of settings.

In addition, I think my experience as a parent of a child with a disability adds a level of connection to parents during the special education research process that others may not experience. This is not to say that people who don't have kids with disabilities can't be effective researchers in the field of special education, they absolutely can, but for myself, I feel more connected to my professional tasks now that I have gone through some of what these families experience themselves. I have always enjoyed working with families, but the work became more meaningful and personal to me, and I felt like I could connect with families more richly than I could before I had a child with a disability and experienced firsthand what they were going through. I would like to believe that families can benefit from this deeper connection, thus increasing the richness of their contributions to research studies.

Flexibility is the number one aspect of the research environment that supported my participation. I absolutely love having a job and role that I can do to the best of my ability while also being able to support my family first and foremost. I also was excited about an opportunity to continue working with families. The hardest part about transitioning from working in a clinical setting to going to a university setting was stepping away from direct service with families and children. Participating in this project has allowed me to do both.

#### Recommendations

I think it's important when conducting field research to involve team members who are invested in the project. They must have an interest in and emotional connection to the work they are doing. Research is a highly controlled process, and the strongest studies will likely have team members who are highly passionate about the topic. I also think that researchers in the field of special education need to be studying things that will have the greatest impact on families. I think a huge part of involving parent professionals in the special education research process is hearing from them what they think needs to be studied more. What do we need to learn in our field that will help families? Where are services lacking and what kinds of things can we as professionals be doing to better the lives of families and individuals with disabilities? Some of the best people to answer those questions and the people who will be the most invested are people who have experienced both sides. These contributions are valuable at all phases of research: in the beginning during the brainstorming process, during the study itself, and in follow up as implications are analyzed, reflections are made, and recommendations for future research are discussed.

Researchers need to be flexible with families and to act in ways that help families feel that their involvement is prioritized. They also need to be organized and clear about their expectations. In the field of special education, we need to be mindful about things like family dynamics and scheduling while also being sensitive to the diverse needs of the individuals with disabilities themselves. Parent-researchers may have a natural ability to do this, as they are used to highly scheduled lives and garnering flexibility when making commitments. When working with families, I touch base with the parents ahead of time to gather information about anything that might help make any of our interactions as smooth as possible. Finding out some basic information about their child, things they like and don't like, their best mode of communication and any potential behaviors ahead of time can really go a long way in being prepared. This strategy can be used as well with parent researchers. Find out what their strengths and areas of interest are to determine their most powerful role on your team before determining roles.

A final suggestion is to welcome all team members equally onto projects. I felt readily welcomed on this project as a team member and was mentored by individuals who had been on the project longer than I had, which really helped me to become involved quickly and effectively. This can help not only with morale between research team members (including parent researchers) but can also contribute to consistency in data collection and more efficacious results.

I highly value research and know that everything we know currently about autism and special education is because someone studied it and then was able to teach it to someone else. There is so much to learn still in our field and without research and willing participants, our learning would halt. My daughter actually received her genetic diagnosis through a research study on genetic causes of autism. Our participation gave us access to medical testing and information that we would have otherwise not been available. For us, the participation in the study was life changing. I am thankful we had the opportunity we did to be involved. This gratefulness contributes to my eagerness to help families feel involved and important in their research participation.

I have had mixed experiences with families and their opinions on research. I have spent time working in lower income areas with families who haven't accessed higher education. Some families are not knowledgeable about the research process and what it entails. Some parents want to have absolutely nothing to do with it, while others value participation and show a desire to share their own situations to potentially help others. Some families may be quiet about their professional skills and may be harder to identify as potential team members. There is merit on all sides and parents should be encouraged to only participate with what they feel comfortable. As a parent researcher myself, I feel that I can help more apprehensive parents understand the process and maybe not be so wary of it. Perhaps involving parent researchers could potentially increase parent participation.

### Researcher With CSESA

When I heard about the CSESA project, I knew immediately that I wanted to be involved. The role I held in the project of interviewing young adults and their parents was so exciting to me. Of the age groups I have worked with, I have had the least amount of experience with young adults and I was so eager to learn more about families who have older children. The project really fit my professional needs as well-the involvement seemed flexible, yet meaningful and allowed me to continue to connect with families. Ideally, I wish I had been involved with the CSESA project from the beginning and been a part of the development process when choosing assessments and instruments.

For me I would have to say that the most impactful part of being involved in autism research has to do with knowledge sharing. I have learned so much from families during research projects and I hope they feel the same way about me. I have learned so much about my own child and her disability both by working with other families and participating in research. It has also been very powerful for me to have been able to connect with two other mothers on this project who are also parent researchers. I have friends with kids, I have friends who have kids with special needs, but I don't have many friends who have kids with special needs that are also professionals in the field. It is a completely different kind of connection and I'm thankful to have these women in my life. For the CSESA project in particular, I can only hope that us working together for our roles on this project helped to empower families and contribute to the success of the study as a whole.

## Yulian

### Personal and Educational History

My parents immigrated from Mexico to the United States the year I was born in 1984. The reason they left Mexico was to provide my two sisters and me the opportunity to have an education and live the “American Dream.” From the time I was a young girl I was encouraged to look into my future and a future profession. However, my parents, as most immigrant families, had many challenges to support a family in the United States.

By the time I was in 1^st^ grade I had experienced trauma and abuse. The school intervened and sent me to counseling as part of the strategies to prevent me from being removed from my parents' home. That is when I realized that I wanted to grow up to help families by becoming a counselor.

By the time I was 21, I completed all the requirements at UCSD for pre-med, but decided to pursue my first dream of helping children and families, so I switched to Psychology. During my last year in my undergraduate program, I worked as a research assistant for the Psychology Department. I would gather data and assist with translations of forms from English to Spanish.

The first year of my graduate program in Marriage and Family Therapy (MFT), I was told that I had a tumor and that I would not be able to have children. Due to the treatment of the tumor, I developed Rheumatoid Arthritis that required me to be on chemotherapy. I did not believe I would be able to have a family, but at 22, shortly after I was married, I was blessed to learn that I was pregnant with my first daughter.

The profession of MFT suited my goal of pursuing a career while being the full-time caregiver to my first born. In 2009, I welcomed my second daughter. In 2010, I decided to go back to school to pursue a Doctoral Degree in Psychology. This was about the same time I started to notice my second daughter was developing differently than my first and took her to get an assessment. At the time, her doctors thought she only needed speech therapy as she was non-verbal. We went from a bilingual home (Spanish/English) to a fully English home thinking this would help my daughter with language development. However, this did not help my daughter and the developmental differences became more apparent.

In 2011, I welcomed my third daughter. I continued to seek help for my second daughter as I continued to notice that my second daughter was not progressing like my first. When she turned 3 years old the district denied me services stating that she did not have a “significant” delay in her learning. They suggested I follow up in 6 months to see if she had progressed. In that moment I felt I had not been listened to or understood and the district had simply brushed me away. I attributed this to being a young, Mexican, new mom who did not know what services to ask for or what the district's responsibility was to my daughter. After this incident, and a few more negative encounters with the school district, I decided to change programs and pursued a Doctoral Degree in Education. I wanted to empower myself to advocate for my daughter with the school districts that had denied her services. I completed my doctoral Degree in Educational Leadership in 2014.

### The Intersectionality of Parent and Professional

I first became pregnant toward the end of my graduate degree so I started my professional journey while trying to navigate being a first-time mom. I tried to manage as best as I could, but balancing being a young entry level professional and young mother was very difficult. When I noticed that my second daughter exhibited many of the characteristics of children with autism I was working as a therapist in a Foster Care Agency. As I had worked in the field and had some experience with using ABA therapy with my clients I was able to start using some of the strategies that I was using with my clients with my daughter.

My role as a mother is my priority. My children are my priority. I only take professional opportunities that support my being able to drop off and pick up my children from school. If an employer or agency is too far from my children's home or school and does not align to my child's schedule, I do not consider it. This can be limiting because I essentially can only work while my children are in school, late evenings after my husband comes from work, or on weekends. It can be really frustrating because I have worked really hard professionally and academically, but I am often not a good candidate for a full-time position. Due to my child's needs it would be wonderful to have medical coverage, sick leave, and other benefits that I unfortunately do not receive as a part time employee.

### Sources of Support and Challenges

I did not have support from my family to support a professional career after a bachelor's degree. According to stereotypical gender roles once I was married and had children it was understood that I would no longer work and that my role would be to “Take care of my husband and children.” When I pursued a doctoral degree, I was shamed and labeled as “Selfish” from my family. They did not help with childcare, especially with my child that had special needs due to her high demands of fleeing and having behavioral challenges. My husband and I would work opposite schedules, and I attended school online and on weekends. I was unable to apply to a traditional doctoral program because of the time constraints that it would have on my family. I found an educational program and teaching/counseling jobs that I could tailor to be opposite of my husband's schedule so that one of us could always be home to care for our children while I studied and/or worked.

As the primary caregiver of my child with special needs, every professional choice is dependent upon being able to care and be available to advocate, support, and be present for their daily challenges. I have declined professional advancement opportunities and have multiple part time positions that fit into my daughter's schedule. My experience in the workplace is often met with frustration due to employers and supervisors not understanding of needing to change my work schedule to attend IEP meetings or meet with an educational advocate. Without my husband's financial, emotional, and parenting support I would have been unable to be a professional and a full-time caregiver of my children. His hands-on parenting enabled me to function as an advocate for my child's needs. Even if he was not part of many of the doctor's appointments or IEP meetings he provided me with the flexibility to be present with my daughter.

I do not have much experience being in a professional field prior to being a mother. What I can say is that I feel that I have never been given the opportunity to have a full-time position in any of the agencies with the flexibility of being able to still support my child who needs my support on a daily basis. This means that other inexperienced applicants have been given the opportunity over me. This has hindered me professionally because it has caused my confidence and professional growth to become stagnant. I hold positions that are beneath my educational and professional experience, but since they work for my family's schedule I take on. There is a higher level of exhaustion and burn out also associated with multitasking family commitments and contractual duties. I often feel as though I am failing both as a parent and as an employee because one affects the other.

### Parent-Professional as Co-researcher

#### Assets and Challenges

My educational background in Psychology and Educational Leadership have provided me with many assets because I have a diverse foundation. I have expertise in Counseling and Education which allows me to have knowledge on Qualitative and Quantitative methodology. Having had experience as a researcher in completing a dissertation with sequential mixed methods research design also enables me to attain information without bias and to honor the ethical guidelines of being a researcher.

Being a mental health clinician and educator and being a parent with a child with disabilities has enabled me to have empathy and compassion to the struggles faced by parents that have children with disabilities. It has allowed me to connect with both my clients and students where I can engage in deeper conversations. I am confident that the empathy and compassion that I possess with working with families that have children with special needs as a parent with special needs far surpasses that of a researcher that is not a parent or a parent with a child with special needs. The research field is created to use student researchers that do not have familial experiences and although they may have traits of compassion it really does not compare to the level of understanding to those who have lived similar experiences.

There is added value in both being a professional and a parent raising a child with a disability based on the ability to connect both personally and through scholarly knowledge to the subject. There is a true subject matter expertise in both the lived experience and the knowledge from the studied theoretical framework. For example, when conducting interviews, the value that is added from having the experience of having a child with disabilities is knowing what sort of clarifying or follow up questions may be asked of the families. Without having a child with autism I would have not known some of the terms, therapy treatment or behaviors that were referred to by the families.

Parents bring a different maturity and lived experience when participating in research. Even now, as compared to 8 years ago when my daughter was first diagnosed, I feel a big difference in the way that I relate and connect with others that have been impacted by having a child with a disability. I believe that this experience, at times, holds more value than the EdD or the LMFT behind my name.

Personally, some of the challenges that I had with being a parent with a child with disability was feeling emotional when parents talked about their challenges and having had similar experiences with my own child. The benefit, however, was that I believe that the parents and young adults were able to see a sincerity to my reactions that enabled them to open up more to share.

#### Barriers to Participation as a Parent/Co-researcher

Schedules are really important for the inclusion of parent researchers. Another important factor is flexibility of doing projects outside of the traditional working environment and schedules. Inclusion in being part of the research design, not just the ones coming in to gather the data. Training and opportunities for growth are also important areas that are limiting due to the lack of flexibility in schedules and demands of being a parent of a child with special needs.

I think the research culture fosters an environment where young, childless, and inexperienced future professionals are given many more opportunities. As a parent who has limitations in her schedule, I do feel that childcare and a traditional working schedule has been my biggest barrier to my participation in research. It is not conducive to my being able to participate as a full time professional and also being able to care for my daughter. It was not an option for me to not be the one caring for my daughter as I did not have family, outside of my spouse, to help with childcare.

### Researcher With CSESA

To be completely honest, I did not believe that I would ever get the opportunity to participate in a large-scale research study like CSESA due to the limitations that I have as a parent. It has been really impactful to be able to see that my voice in the field has meaning and value. The benefits of participating as a researcher is being able to see through the lens of a parent with a child of disability and noticing some of the areas of weakness in both my advocacy for my own child and in the research design of the research study.

I valued the role that the current research has had in influencing change in the community and families of children with disabilities. I felt that in this way I could catch a glimpse of how I could support my daughter when she reached high school. I also thought it was a great opportunity to learn of resources and information available. I believe it is hard for parents to typically see the value in research because it is not something that is public knowledge for people to see that research is a catalyst for change in the lives of families of children with a disability. Especially when the incentive is low and they are not motivated by intrinsic needs of helping future individuals and families with disabilities. To be part of a study that empowers young adults to overcome the challenges that autism can bring and highlights to families the resources and focuses on the resiliency of both parents and young adults has been a true blessing to witness as it has given me hope for my daughter in the future.

## Interpretation and Discussion

The parent/co-authors were asked to write their personal stories considering any biases, obstacles, perceived assets, and supports they experienced in their roles as parents/mothers and professionals separately as well as to consider the intersectionality of these two roles. The main themes identified in the autoethnographies include: prioritizing children and professional sacrifices; professional training as asset for parents; potential bias toward parents in professional contexts; assets as parent-professionals; and obstacles for maintaining the intersectional roles of parent and professional. Each of these themes are elaborated upon with examples from the autoethnographies and connection to previous literature on parent-researchers or the inclusion of parents on research teams.

### Prioritizing Children and Professional Sacrifices

Each of the autoethnography authors clearly stated that their priority was their family and ensuring their child with autism was receiving good care and quality education to meet their needs. The three parent-researchers discussed an awareness of the resources that this takes to be able to care for family while meeting any employment obligations. They all stated they were fortunate to have husbands and families who provided support, and that they were in a financial position to have funds to pay for outside support if needed. They discussed that these resources were not available to everyone and recognized that other families face greater struggles than they have. When outside support was needed it was important that the carer had competence beyond the typical high school student sitter or typical respite worker (e.g., had training to address epilepsy or understand dietary restrictions). Many families do not have access to this type of care and/or are not connected to resources that provide financial support to help with this need.

Each of the parent-researchers made professional sacrifices because family is priority. In most cases the lack of employment positions with flexibility, and lack of employers with an understanding of the parent's responsibilities, resulted in; (a) obtaining part-time employment, (b) remaining in a position longer than others in the organization, (c) being passed up for promotions, and/or (d) receiving a lower salary. In addition, one co-author has been delaying obtaining a doctoral degree. Although our US society has changed from the time when women were asked to leave their positions as soon as they were married so they could prioritize husbands and family, these parent-researchers experienced clear obstacles to maintaining and advancing positions in their organizations and agencies and maintaining family as priority. Systems of support for professionals who also are parents of children with significant needs are far from a workplace standard.

### Professional Training as Asset for Parents

Each of the parent-professionals described the impact that the knowledge obtained in their professional education, on-the-job training, and professional experiences had on the quality of services their child received. They knew their rights as parents in the Individualized Education Plan process and were aware of the components of a high quality and evidence-based program along with the criteria for a well written and meaningful goal. They also discussed the benefits of knowing how to navigate the educational, social, and behavioral service systems. In fact, both Natalie and Yulian made choices to obtain degrees that would enhance their ability to provide advocacy for their child's services. Natalie and Gretchen discussed how much they valued sharing this important competence with other parents. These parent-researchers stated that professional training was empowering and sharing advocacy skills to empower others is rewarding. They commented that professional choices they made involved consideration of the benefits for them professionally as well as personally as a parent of a child with autism.

### Potential Bias Toward Parents in Professional Contexts

Each of the parent-researchers experienced some biases toward parents in professional and educational contexts. Natalie stated that before she would reveal she was a parent she would hear teachers or future special educators describing the challenges of working with “high-profile” parents and even some university professors would speak negatively of parents as a group. She elaborated that instead of proudly sharing with those professional colleagues who are autism researchers that she brings experience as a parent of a child with autism to her work, she shared her concerns that her colleagues would think less of her and value her opinions and contributions to a project less. She holds this feeling to this day. These highly competent professionals wondered if they are taken less seriously by colleagues when they become aware that they are also parents of children with autism.

Morris et al. (6) describe the importance of supporting families so that they feel valued as part of the research team and assisting parents to move away from initiating input with the phrase, “I know I am only a parent but…” Even if parents feel confident in the quality of their input the co-authors wondered if there was not an assumption from researchers that parents are not able to approach their work with the same objectivity as the other team members. The co-authors discussed the possibility that parents were not sought to join research teams because researchers held beliefs that parents of children with autism may have more of a bias than non-parent researchers and may not be able to maintain the objectivity needed in research. Evaluating this possible researcher bias in future studies would help to determine if this impression is accurate so that the issue can be addressed if a bias exists. It also could be true that all researchers bring some biases and personal perspective to the research even as they aim to develop measurement systems and procedures that are as objective as possible. It may be important for researchers to address the potential issue of individual biases from a research team by purposely seeking various perspectives during the design, implementation, and data analysis phases of the research.

### Assets as Parent-Researcher

All the parent-researchers agreed that “there is no such thing as spare time for us.” If they are hired as part of a research project it is because they value the focus of the research and they make a commitment to the work. They stated, “We get it” and they will do what it takes to get the job done. They do not just work nine to five. If a study involves interviewing parents the parent-researchers can work around parents' schedules. If parents wanted to meet on weekends they were able, and in fact, preferred to meet at this time. Yulian stated that being involved in research is more than a job, or professional development experience. We realize that the research has the potential to change the lives of their children. Parent-researchers also can help determine if the proposed research outcomes are really of value to families. Having someone who knows about the impact on families can be helpful to ensure that the research meets this, often stated, goal.

In addition, these parent-researchers felt they could easily develop a rapport with parents who were participants in research. Gretchen stated that she is able to empathize with parents as they shared experiences during interviews that were part the data collected for the CSESA research. Yulian stated that she believes that parents feel comfortable with her because she has had similar experiences even if she does not share these experiences directly. Natalie stated that when she interacts with other parents of children with autism, they inspire her to think broadly about potential possibilities for other children with autism.

Consistent with the point made by authors included in Shen et al. (9) scoping review of practices for engaging parents as co-researchers, the co-authors in this article agree that parent-researchers bring benefits such as making messaging more meaningful to parents, optimizing research timing, maximizing participation involvement and attendance, and through their passion and enthusiasm, motivating other research team members. In addition, several authors describe empowering parent-researchers as a result of this shared process (9). The use of parent-professionals that are from the communities where research is being conducted could have the added benefit of focusing on what is needed by families in that community. Magaña et al. (21, 22) used mixed methods and a randomized waitlist-control group design to evaluate the benefits of a Community Health Worker Model or Promotora de Salud Model where Latina parents indigenous to the community were prepared to deliver the content of an education intervention for parents of children with autism that resulted in increased knowledge about autism, use of evidence-based practices, and understanding of rights and advocacy skills.

### Obstacles for Maintaining Intersectional Roles of Parent and Researcher

The obstacles to obtaining positions on research teams consistently described by the co-authors included a lack of flexibility in work schedules to address family needs, insufficient pay to compensate for the quality of the childcare needed by their children with autism, and a lack of valuing of the parent perspective from research team members. Hackett (8) describes parents who participate as co-researchers as a self-selecting group due to the time and commitment required over a sustained period. There is much research teams can do to seek out and encourage participation from parents of children with autism who have the professional competence that qualify them for employment. This is likely to happen, only if the contributions made by those who are parents of children with autism are truly valued by team members.

## Implications and Recommendations for Autism Researchers

The consistent message from the parent-researchers and co-authors of this article is that there is added value to a study that employs individuals with the intersectional roles of parent and professional in a related field. An indication that the competencies and lived experiences of parent-researchers are valued would occur when these individuals are sought to fill positions on research teams. Similar to other authors (6, 7, 23), all three parent-researchers felt it was important to involve parents in the research design and the development of the materials/questionnaires. Vander Stoep et al. (7) state that parents who are community partners should have real influence on the project direction and be part of the analysis and interpretation of data along with how the results are distributed. Measuring the impact of the autism research on families also would be important. It may benefit the field to consistently determine how the research impacts families, and perhaps this needs to be standard consideration of all research projects. Measuring the impact of autism research on families will help to determine which lines of inquiry are not impacting families and which may benefit from further replication to maximize the research with important impact.

The following recommendations were identified as helpful when including parent-professionals as part of the research team: Clearly identify the role and expected time commitment for the position; ask parent-researchers what they need to participate; provide a salary similar to other members of the team; and pay for mileage and drive time if relevant. The parent-researchers agreed that if they get paid less than the cost of childcare they are basically working for free. If budgets allow and the project can pay for respite care this will mean the parent-researcher can be more flexible to take time away from family.

### Beyond a Symbolic Gesture

Multiple co-authors described situations when they were participating in meetings with researchers or task group members and felt like their input was not valued. They described being invited to attend meetings or task forces and realizing they were there as a symbolic gesture. This becomes clear when contributions to the conversation are ignored. The co-authors discussed the importance of making sure that parents are truly valued for what they bring to the research team.

The co-authors also discussed that it would be important that any flexibility provided to the parent-researcher is perceived by the team as important in order to obtain their added value. The parent-researchers described previous experiences when the arrangements and flexibility made by employers in order to address childcare needs were viewed by colleagues as providing preferential treatment. Yulian recounted that when she was in her doctoral program she had the impression that her cohort peers thought she got away with having to do less because of the responsibilities she had caring for her children. As a result, she felt like she had to hide that she was a parent, taking care of her childcare needs without discussing this with others, and instead of doing less she felt she had to put in 110% to prove that she was as committed as her peers. There was even a discussion regarding the participation in writing this article and whether the authors' research would have diminished value compared to other research if the reader is aware that the research was completed by parent-professionals, even ones with a doctorate.

In order for the field to benefit from the talents, competencies, and experiences of parent-professionals it would be necessary to have the support systems in place to allow time to devote to professional activities such as employment on a research team. If respite care personnel were better prepared to address the complex needs of some individuals with autism, this would result in increased opportunities for participation in research by more parents. Perhaps a cost-benefit analysis of increasing the training and compensation for respite and childcare services for individual with autism would uncover the benefits of employing parent-researchers. Additional recommendations for future research include the publication of more voices of parent-researchers through interviews and focus groups to determine the extent of the shared experiences and the perceived contributions that can be made to research by individuals with these intersectional identities.

## Conclusion

The three parent-co-researchers sharing this article are each competent professionals with master's and Doctoral degrees in fields of study (education, counseling, & special education) and with professional competence (special educators, behavior analysts, & marriage and family therapists) related to autism research. Considering the current prevalence of autism as 1 in 54 (24) who each have parents, there are many parents of children with autism who are also competent professionals in fields relevant to autism research. If research teams value the contributions of parent-researchers and can create the working conditions to support their participation, they are likely to find suitable individuals to hire. In addition, if there was a clear career path for parent-researchers there may be more individuals with this intersectionality to fill positions on research teams. One of the challenges that remains includes a plan of action to change systems to enable participation by parent-researchers, a topic rarely directly addressed.

## Data Availability Statement

The original contributions presented in the study are included in the article/supplementary material, further inquiries can be directed to the corresponding author.

## Ethics Statement

The studies involving human participants were reviewed and approved by SDSU Institutional Review Board (IRB). The patients/participants provided their written informed consent to participate in this study. Written informed consent was obtained from the individual(s) for the publication of any potentially identifiable images or data included in this article.

## Author Contributions

The order of authorship was determined by contribution to the manuscript. LH conceived of the research focus, selected the method, invited the co-authors to participate and took the lead on writing the introduction, and discussion sections. All authors contributed equally to identifying the focus of the literature search, developing the probe questions for the autoethnographies, identifying the themes and interpreting the data. In addition, all 2nd through 4th authors wrote their autoethnographies. NH organized the literature into a google drive. NH and GG read provided feedback on early drafts of the manuscript and wrote annotated notes on the content. All authors contributed to the article and approved the submitted version.

## Funding

Preparation of this article was supported by Grant Nos. R324C120006 and R325A180091 from the Institute of Education Sciences, U. S. Department of Education. Opinions expressed are not necessarily those of the funding agency.

## Conflict of Interest

The authors declare that the research was conducted in the absence of any commercial or financial relationships that could be construed as a potential conflict of interest.

## Publisher's Note

All claims expressed in this article are solely those of the authors and do not necessarily represent those of their affiliated organizations, or those of the publisher, the editors and the reviewers. Any product that may be evaluated in this article, or claim that may be made by its manufacturer, is not guaranteed or endorsed by the publisher.

## Footnotes

^1^The co-authors acknowledge the preference for the use of autistic as a descriptor by self-advocates and colleagues promoting ableist language. We decided to use person first language for this article as some of the children of the parent-co-authors could be described by multiple categories in addition to autism.
